# Sonographic appearance of syphilitic induration mimicking squamous cell carcinoma in the lower lip: a case report

**DOI:** 10.1186/s13256-020-02547-x

**Published:** 2020-11-04

**Authors:** Satomi Asai, Akihiro Kaneko, Tsukumi Matsuda, Noboru Takanashi, Mika Doi, Haruyo Atsumi, Go Ogura, Naoya Nakamura, Hayato Miyachi

**Affiliations:** 1 Department of Laboratory Medicine, Tokai University School of Medicine, 143 Shimokasuya, Isehara, Kanagawa 259-1193 Japan; 2 Department of Dentistry Oral Surgery, Tokai University School of Medicine, Isehara, Kanagawa 259-1193 Japan; 3 Clinical Laboratory Center, Tokai University Hospital, Isehara, Kanagawa 259-1193 Japan; 4 Department of Pathology, Tokai University School of Medicine, Isehara, Kanagawa 259-1193 Japan

**Keywords:** Case report, Color Doppler ultrasonography, Dental clinic, Lower lip, Pressure test, Syphilis, Ultrasonography

## Abstract

**Background:**

Syphilis is a sexually transmitted disease caused by the spirochete *Treponema pallidum.* Recently, its incidence has been increasing worldwide. We encountered a young woman who presented with induration mimicking squamous cell carcinoma in the lower lip, without major medical conditions.

**Case presentation:**

A 25-year-old Japanese woman presented with a 1-month history of a painless induration in her lower lip. Because squamous cell carcinoma was suspected, a preoperation work up was performed, including laboratory tests, an ultrasonographic examination, and a biopsy. The ultrasonography findings showed an oval-shaped 17 × 11 × 12 mm tumor-like lesion with heterogeneous internal echo and an indistinct border. A pressure test and color Doppler sonography revealed that the lesion was soft with a very abundant blood flow. These findings suggested the possibility of underlying inflammatory causes rather than a neoplastic tumor. Serology tests for syphilis, including the anti-*Treponema pallidum* antibody and reactive rapid plasma reagin tests, were positive. The biopsy revealed no malignancy. Finally, she was diagnosed as having primary syphilis and treated with amoxicillin for 28 days. The rapid plasma reagin value gradually decreased and the initial induration in her lower lip disappeared.

**Conclusion:**

This case highlights the need for prompt examinations for possible underlying infective causes, such as syphilis, when seeing a painless induration with ulcer in the lip. Ultrasonography was helpful in the differential diagnosis of a tumor-like lesion and should be included in addition to syphilis serology tests, such as anti-*Treponema pallidum* antibody and rapid plasma reagin tests.

## Background

Syphilis remains a major public health problem. Recently, its incidence has been increasing worldwide [1]. In primary syphilis infections, patients develop oral lesions which mostly occur on the lip. Additional mucous patches, mainly located on the tongue, occur in conjunction with secondary syphilis [2, 3]. Primary infection of an oral lesion includes chancre, painless tonsillar enlargement, and painless lymphadenopathy [4]. Ultrasonography is known to be useful in diagnosing tumorous lesions of the head and neck, such as the submandibular glands and lymph nodes [5–9]. Some cases of primary syphilis with initial induration or chancre have been reported [10, 11]. A painless ulcer with an indurated margin and a clean base is known as a “chancre.” However, the ultrasonographic findings of syphilis lesions, such as induration or chancre, are not fully understood.

We experienced a case in which ultrasonography was useful in the differential diagnosis of syphilis with an indurated lesion in the lower lip. Here we report the sonographic findings, the clinical diagnostic process, and treatment for patients with atypical induration lesions of the lip.

## Case presentation

A 25-year-old Japanese woman was referred to the Department of Oral Surgery of Tokai University Hospital with an intractable, sclerotic lesion in her lower lip that was thought to be a malignant tumor. She had been healthy until she noticed a painless nodule in her lower lip 4 weeks ago. She visited a local dental clinic and had been diagnosed with cheilitis by bite trauma of the lip. Antimicrobials were not prescribed.

**Fig. 1:**
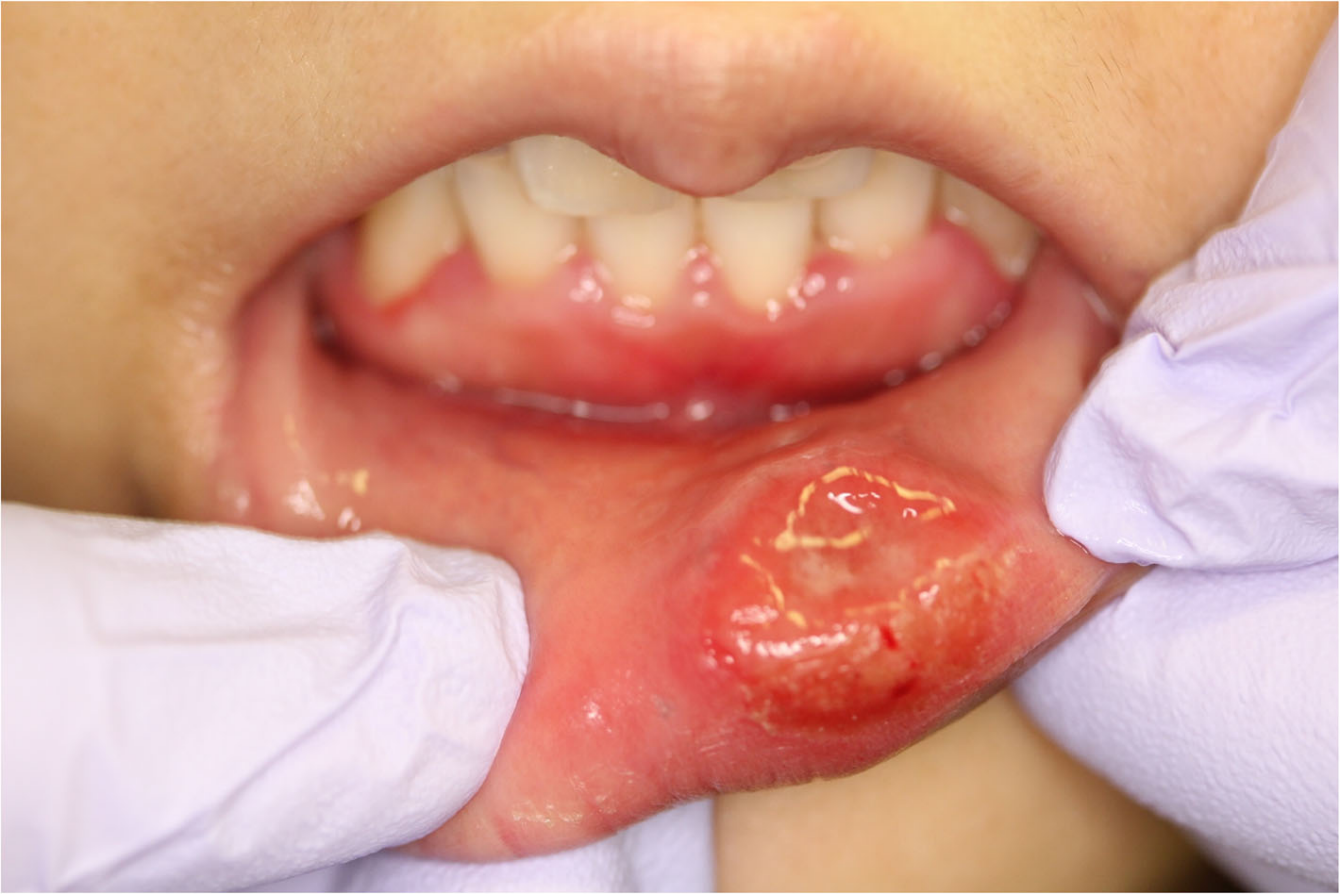
A photograph of the lower lip lesion of a 25-year-old woman with syphilis. A healthy 25-year-old woman presented with a 4-week history of a painless, ulcerative intractable lesion with an indurated margin on her lower lip

She had no history of tobacco smoking or alcohol use. She reported a history of unprotected orogenital contact with a steady male partner approximately 3 weeks before the onset of a painless tumor. A physical examination revealed that she had a nodule in her lower lip, which was approximately 2.0 cm in diameter. It had an ulcer-like lesion in the center and a slightly elevated erythematous and indurated margin (Fig. 1). Further examinations, including genital and rectal examinations, revealed no evidence of malignancy. Fever or apparent cervical lymphadenopathy was absent.

A complete blood count (CBC) and biochemical tests were within reference ranges, except for an elevated C-reactive protein level of 6.48 mg/dL (reference value < 0.30 mg/dL). The blood tumor markers squamous cell carcinoma (SCC) antigen and carcinoembryonic antigen (CEA) were not elevated. Tests for human immunodeficiency virus (HIV) I/II antibodies, hepatitis B (HB) antigen, and hepatitis C virus (HCV) antibodies were negative; these test results were additionally confirmed to be negative 1 month later.

**Fig. 2:**
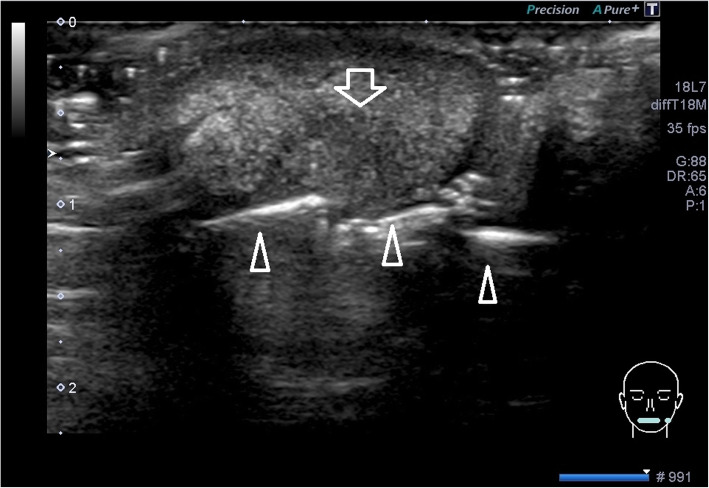
Sonogram of the lesion on the lower lip. A gray-scale and transverse sonogram of the lesion obtained with the patient’s mouth closed. The shape of the lesion was almost oval. A heterogeneous tumor-like lesion of 17 × 11 × 12 mm in size was observed in her left lower lip. The ulcerative lesion was hypoechoic (*white arrow*). The mandibular teeth are indicated with *white arrowheads*

**Fig. 3:**
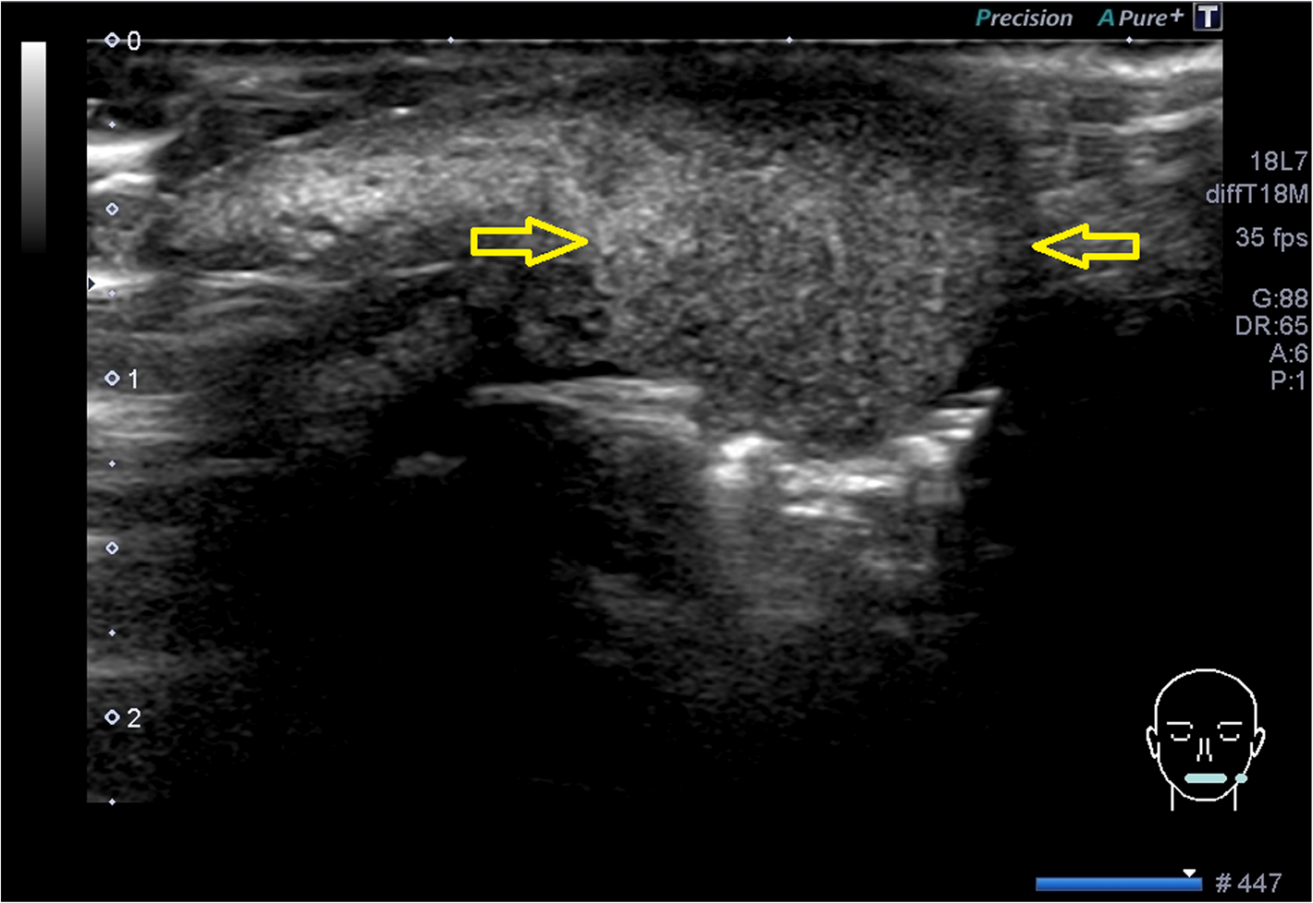
The border of the tumor-like lesion. The border of the lesion was indistinct (*yellow arrows*)

**Fig. 4:**
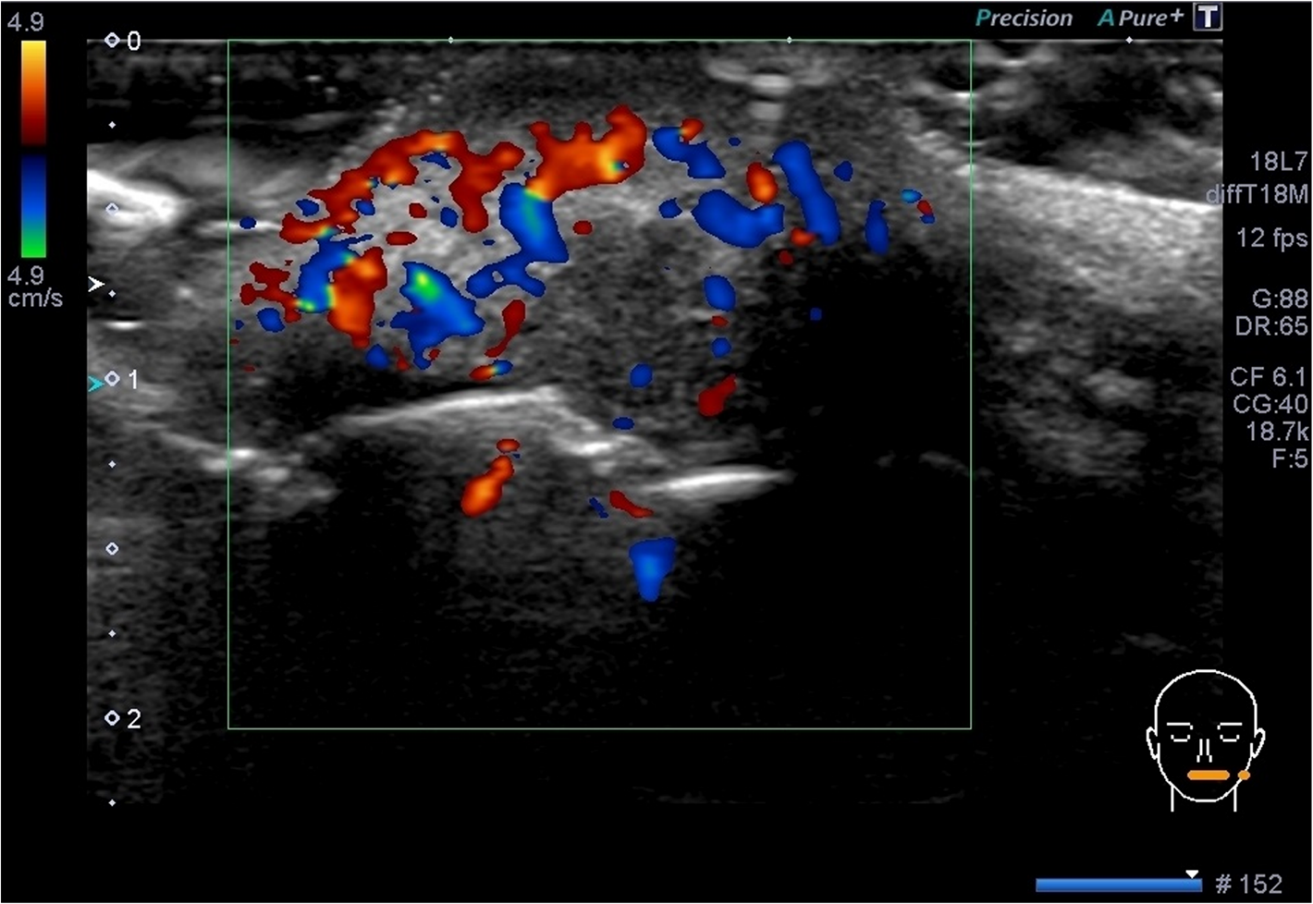
A color Doppler sonogram of the tumor-like lesion. A color Doppler sonogram of the tumor-like lesion revealed very abundant blood flow in the heterogeneous sites and poor blood flow in the hypoechoic part of the tumor-like lesion

An ultrasonographic examination was performed to determine the invasion range of the mass. The examination was performed by a well-trained sonographer (who was specialized in ultrasonography and was licensed as a special sonographer) using an Aplio instrument equipped with a high-resolution 7–14 MHz linear-array transducer PLT-1204BT (TOSHIBA; Tochigi, Japan). The lesion revealed an oval-shaped tumor measuring 17 × 11 × 12 mm in size, with heterogenous internal echo (Fig. 2.) and unclear border (Fig. 3). Color Doppler sonography showed very abundant blood flow signal, except for in the center of the tumor (Fig. 4). Pressure testing with a probe revealed that the tumor was soft, suggesting an inflammatory mass, rather than neoplastic. It was not possible to perform multiple resonance imaging (MRI) because one of her front teeth had a metal crown.

The ultrasonographic findings taken together with a history of unprotected orogenital contact with a steady male partner and a social background with an increased prevalence of syphilis among young women in Japan prompted us to make a differential diagnosis for a syphilitic mass.

Syphilis serology tests were added following ultrasonography. A quantitative antibody test for *Treponema pallidum* (anti-T. *pallidum* antibody) was high at 115.1 U/mL (reference value, < 5 U/mL). A reactive rapid plasma reagin (RPR) test was strongly positive with a titer of 2060 RU. A fluorescent treponemal antibody-absorption (FTA-ABS) test was strongly positive (1:320).

**Fig. 5:**
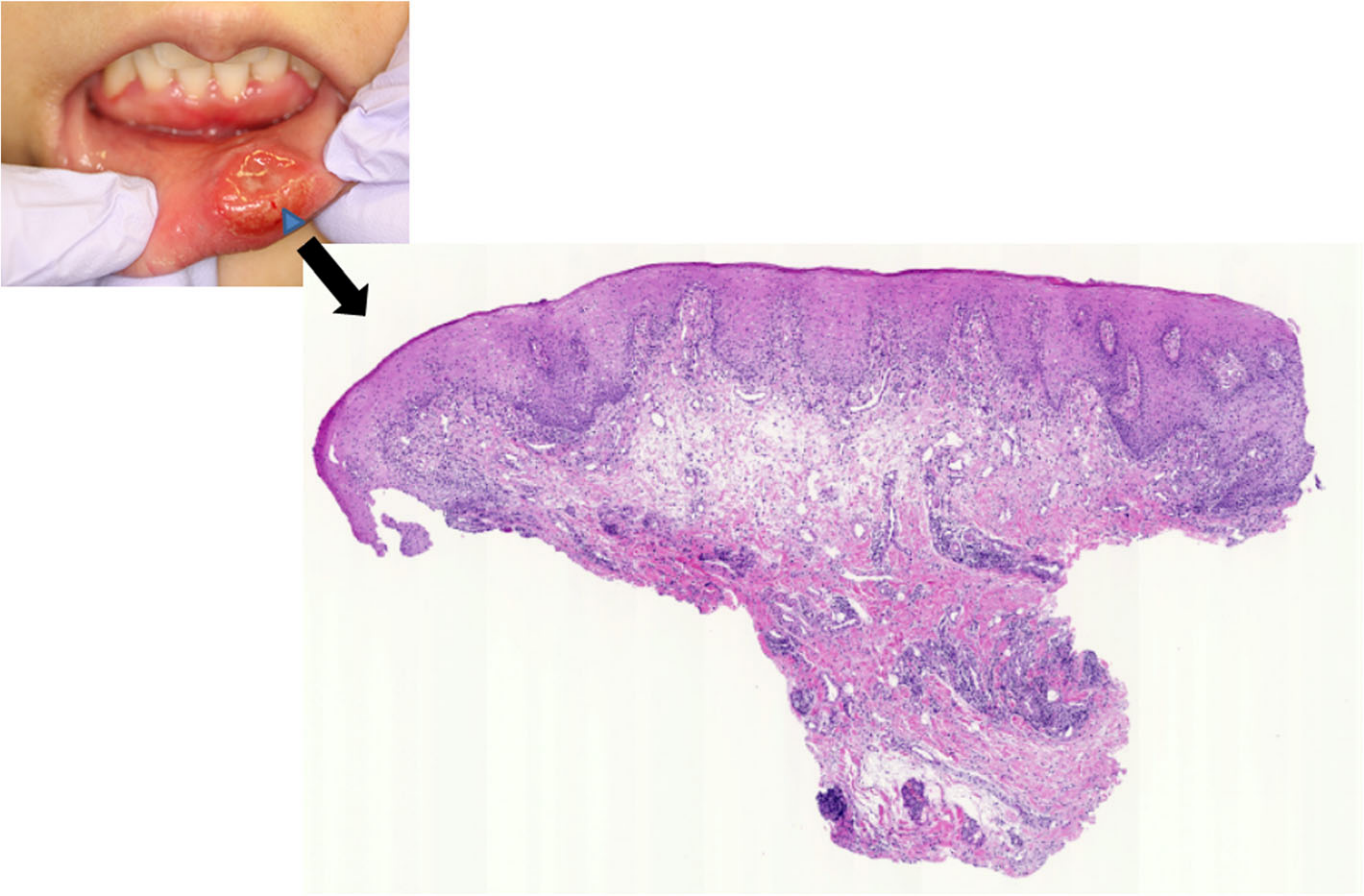
A loupe image of the histopathological examination. The histopathological examination (*blue arrow head*) showed lymphoplasmacytic infiltration around the vessels and appendages in the subepithelial region. No evidence of malignancy was observed

A biopsy of a small part of the sclerotic lesion was performed. A histopathological examination showed lymphoplasmacytic infiltration around vessels and appendages in the subepithelial region (Fig. 5). *T. pallidum* was not found by using Warthin–Starry staining and immunostaining.

**Fig. 6:**
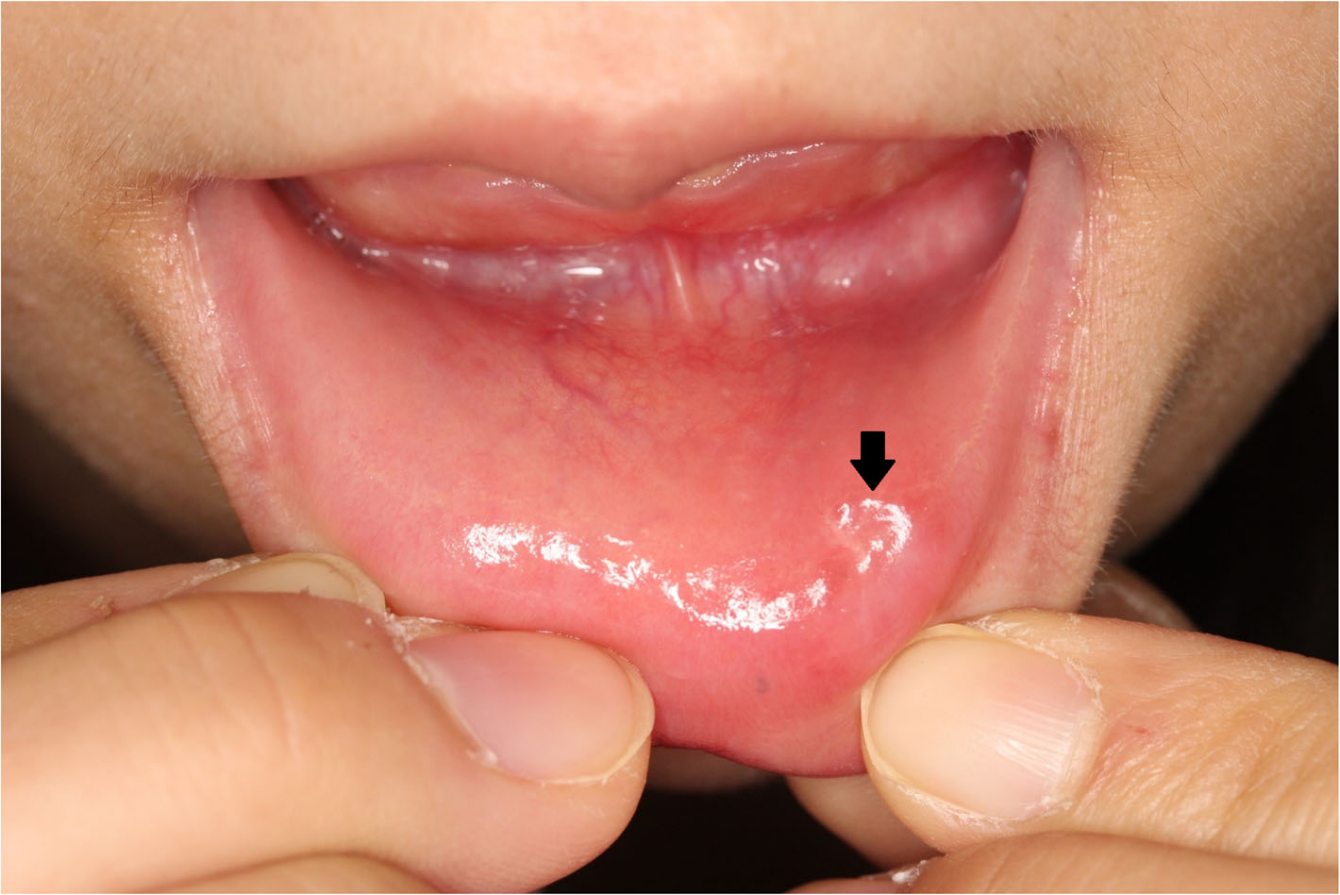
A photograph of the lower lip lesion after treatment with amoxicillin. Her lip lesion had almost disappeared after 3 months (*bold arrow*)

**Fig. 7:**
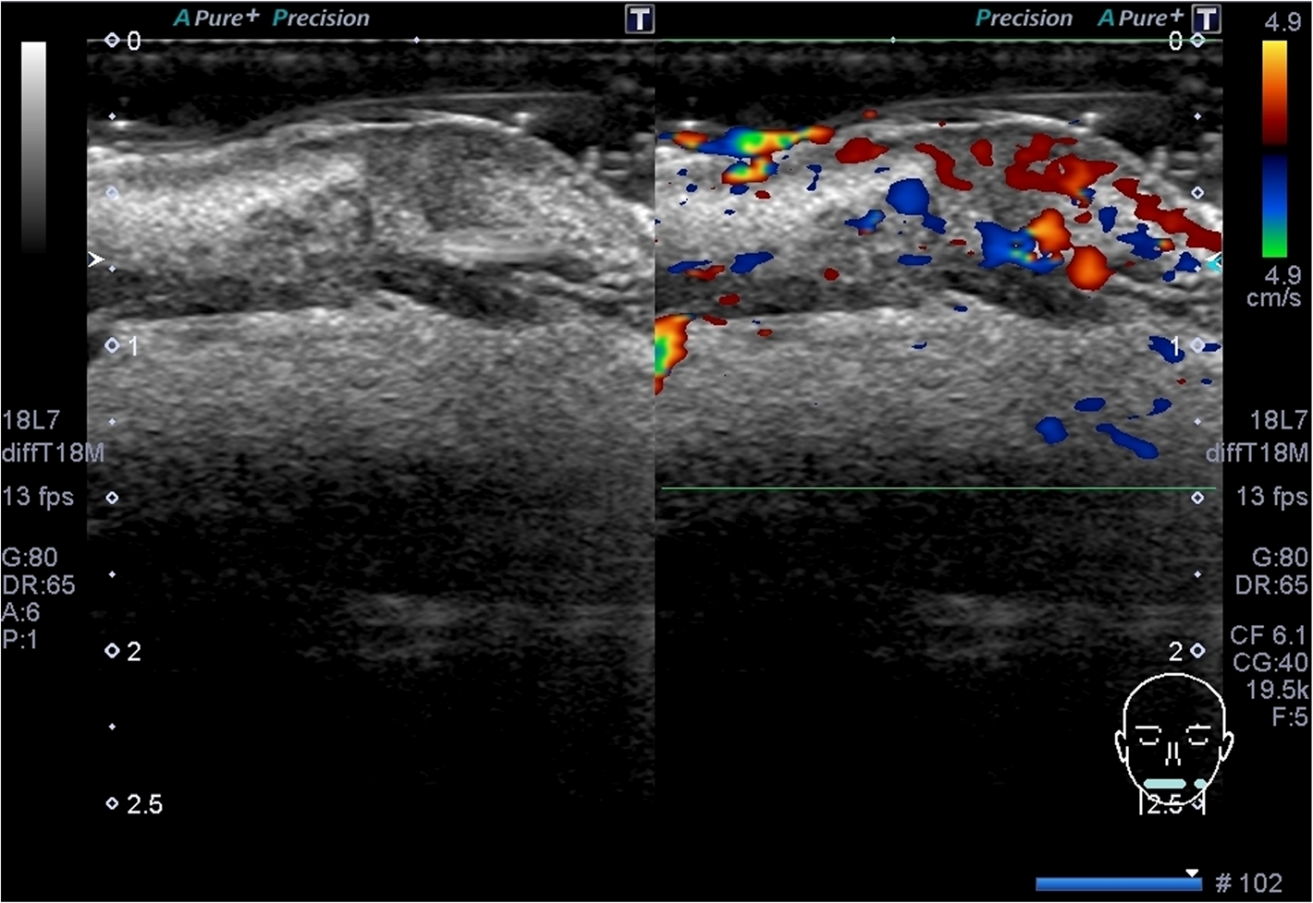
Sonograms of the lesion on the lower lip after treatment with amoxicillin. A gray-scale transverse sonogram (*left*) and color Doppler sonogram (*right*) of the lesion at 3 months after the initiation of treatment. Her lip lesion showed remarkable improvement at 3 months after treatment initiation, with only a hypoechoic lesion with blood flow signals remaining

**Fig. 8:**
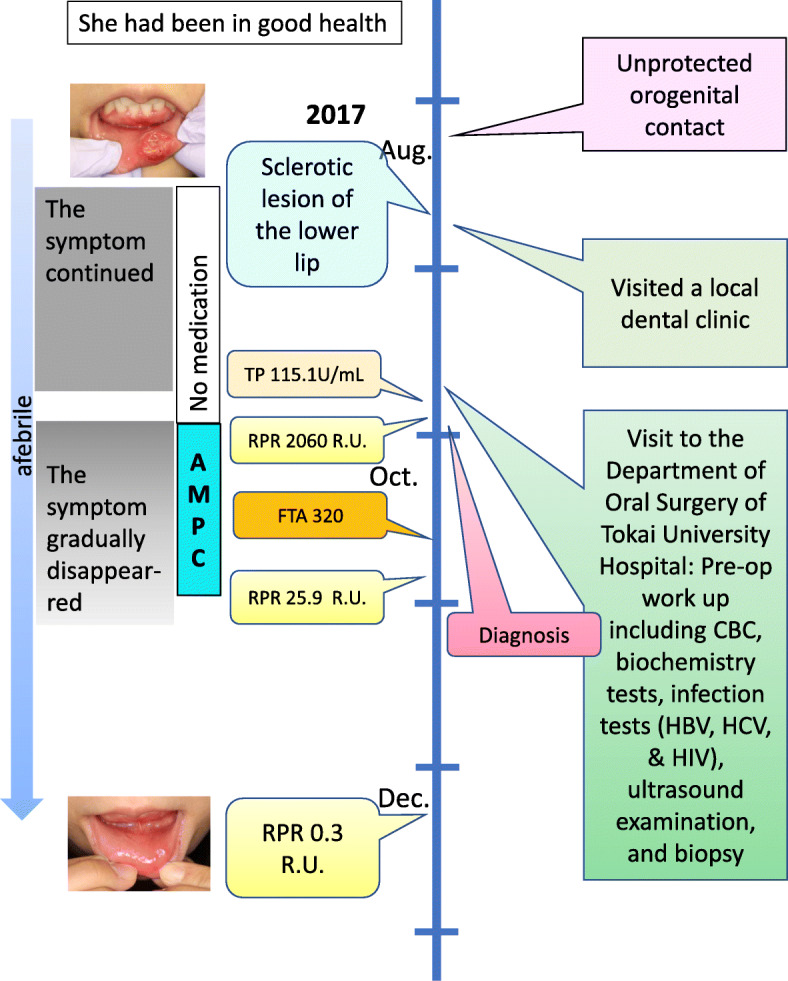
Patient timeline from unprotected orogenital contact to gradual reduction of the lesion. *AMPC* amoxicillin, *CBC* complete blood count, *FTA* fluorescent treponemal antibody, *HBV* hepatitis B virus, *HCV* hepatitis C virus, *HIV* human immunodeficiency virus, *RPR* rapid plasma reagin, *TP Treponema pallidum*

A diagnosis of syphilis was made, with lower lip induration as the primary lesion. Amoxicillin (AMPC) 1500 mg was administered orally for 4 weeks according to the guidelines of the *Japanese Journal of Sexually Transmitted Infections* [12]. Her lip lesion had almost resolved after 3 months (Figs. 6 and 7). She did not show antibacterial side effects. An RPR test became negative with a titer of 0.3. Her sexual partner, who had multiple partners and engaged in casual sex, was also diagnosed as having genital syphilis and received treatment from another hospital. Our patient’s timeline is shown in Fig. 8. This study was approved by the Ethics Review Board of Tokai University (18R-192).

## Discussion and conclusions

We encountered a case of a 25-year-old Japanese woman who had been referred for potential SCC and presented with syphilitic initial induration of the lip. The syphilitic infection in the present case was confirmed by a positive serological test result for RPR, anti-T. *pallidum* antibody, and FTA-ABS. In combination with the ultrasonographic findings, a diagnosis of a syphilitic lesion of the lip was made. An initial induration of syphilis is a painless nodule with an ulcer that most commonly forms during the primary stages of the disease. Initial induration develops 1–3 months after infection and has an extragenital presentation in 12–14% of patients with primary syphilis; the oral mucosa is the most frequent location as a consequence of oral contact [1]. History taking in our patient suggested that her initial induration was transmitted through unprotected orogenital contact.

The differential diagnosis for initial induration of the lip includes cancer, traumatic ulcer, aphthous stomatitis, chancroid, herpes simplex, tuberculous chancre, and deep mycoses (for example, actinomycosis, Wegener’s granuloma, and Behçet syndrome) [11, 13]. As a painless tumor, differential diagnosis could possibly exclude traumatic ulcer, aphthous stomatitis, herpes simplex, deep mycoses, and Behçet syndrome. As a painless mass, differential diagnosis included cancer, chancroid, and tuberculous chancre.

Cancer of the lip is the most common malignant tumor affecting the head and neck. The most prevalent histological tumor type found in lip cancers is SCC, followed by basal cell carcinoma. A non-healing ulcer is the most common physical presentation. Early lesions can be subtle and appear as flat, discolored areas (for example, leukoplakia or erythroplakia). The atypical painless ulcer of the lip in our patient required a differential diagnosis besides cancer such as SCC. An SCC lesion may show an abundant blood flow signal on color Doppler sonography, as was seen in our patient. SCC was excluded based on the findings of a pressure test using a probe. Typically, SCC is a firm tumor that does not transform on the pressure test and does not show a rich blood flow signal [14, 15].

Tuberculous chancre is very rare, and occurs at the site of inoculation in unsensitized individuals. The lesion begins as an asymptomatic papule at the site of injury, which later ulcerates. Any area experiencing chronic irritation or inflammation is predisposed to localization of *Mycobacterium*. In this patient, tuberculous chancre was unlikely, since there was no preexisting trauma [16]. In order to appropriately exclude tuberculous chancre, performing an interferon-gamma releasing assay (IGRA) is required for the diagnosis of a tuberculous infection.

The diagnostic process for identifying syphilitic initial induration when oral lesions are encountered in daily clinical practice is critical, due to its increasing incidence and the multifarious routes of transmission by sexual intercourse, including oral transmission. Ultrasound examinations are non-invasive, easy, and prompt [5–9]. Furthermore, pressure testing using a probe to investigate the elasticity of a lesion is useful for differentiating tumors of superficial organs [17].

The present case indicates that when patients present with chronic indurated tumors with an ulcer in the lip, physicians and dentists should consider the possibility of underlying infective causes, such as syphilis. Ultrasound examinations should be considered a part of the routine diagnostic process.

## Data Availability

Not applicable.

## Abbreviations

AMPC: Amoxicillin
CBC: Complete blood count
CEA: Carcinoembryonic antigen
HB: Hepatitis B
HCV: Hepatitis C virus
HIV: Human immunodeficiency virus
IGRA: Interferon-gamma releasing assay
FTA-ABS: Fluorescent treponemal antibody-absorption
MRI: Multiple resonance imaging
RPR: Rapid plasma reagin
SCC: Squamous cell carcinoma
